# Beyond Binding: The Outcomes of Antibody-Dependent Complement Activation in Human Malaria

**DOI:** 10.3389/fimmu.2021.683404

**Published:** 2021-06-08

**Authors:** Dilini Rathnayake, Elizabeth H. Aitken, Stephen J. Rogerson

**Affiliations:** Department of Infectious Diseases, Peter Doherty Institute for Infection and Immunity, University of Melbourne, Melbourne, VIC, Australia

**Keywords:** malaria, immune complexes, classical complement pathway, infected erythrocytes, complement regulatory proteins, *Plasmodium falciparum* erythrocyte membrane protein 1

## Abstract

Antibody immunity against malaria is effective but non-sterile. In addition to antibody-mediated inhibition, neutralisation or opsonisation of malaria parasites, antibody-mediated complement activation is also important in defense against infection. Antibodies form immune complexes with parasite-derived antigens that can activate the classical complement pathway. The complement system provides efficient surveillance for infection, and its activation leads to parasite lysis or parasite opsonisation for phagocytosis. The induction of complement-fixing antibodies contributes significantly to the development of protective immunity against clinical malaria. These complement-fixing antibodies can form immune complexes that are recognised by complement receptors on innate cells of the immune system. The efficient clearance of immune complexes is accompanied by complement receptor internalisation, abrogating the detrimental consequences of excess complement activation. Here, we review the mechanisms of activation of complement by alternative, classical, and lectin pathways in human malaria at different stages of the *Plasmodium* life cycle with special emphasis on how complement-fixing antibodies contribute to protective immunity. We briefly touch upon the action of anaphylatoxins, the assembly of membrane attack complex, and the possible reasons underlying the resistance of infected erythrocytes towards antibody-mediated complement lysis, relevant to their prolonged survival in the blood of the human host. We make suggestions for further research on effector functions of antibody-mediated complement activation that would guide future researchers in deploying complement-fixing antibodies in preventive or therapeutic strategies against malaria.

## Introduction to Malaria

Malaria remains one of the major causes of severe morbidity and mortality globally. In 2019 alone, there were 229 million clinical episodes of malaria causing 0.4 million deaths. The most vulnerable groups include children under five years of age and pregnant women and the heaviest burden of disease is concentrated in sub-Saharan Africa (1). Clinical malaria presents as a febrile illness, that can progress to severe disease, causing death (2). Severe malaria often manifests as severe anaemia, cerebral malaria or acute lung or kidney injury, Lung or kidney injury may lead to pulmonary oedema or renal failure, which is less common in children than adults [reviewed in (3)]. In pregnant women, infection in the placenta may cause adverse outcomes including abortion, stillbirth, intrauterine growth retardation, low infant birth weight, and neonatal death [reviewed in (4)]. Treatment strategies involve the use of artemisinin combination therapies, while vector control and effective surveillance are also important [reviewed in (5)].

In people living in malaria-endemic areas, immunity to malaria is gradually acquired following repeated exposure so that over time individuals become relatively protected from malaria and its complications [reviewed in (6)]. This naturally acquired immunity was demonstrated to be antibody-mediated, when antibodies transferred from apparently immune adults to young children with clinical malaria were able to reduce parasite densities (7).

The leading malaria vaccine candidate RTS,S induces antibodies to the circumsporozoite protein, and both naturally acquired and vaccine-induced antibodies fix complement and engage Fc receptors (8–10), discussed later.

**Figure 1:**
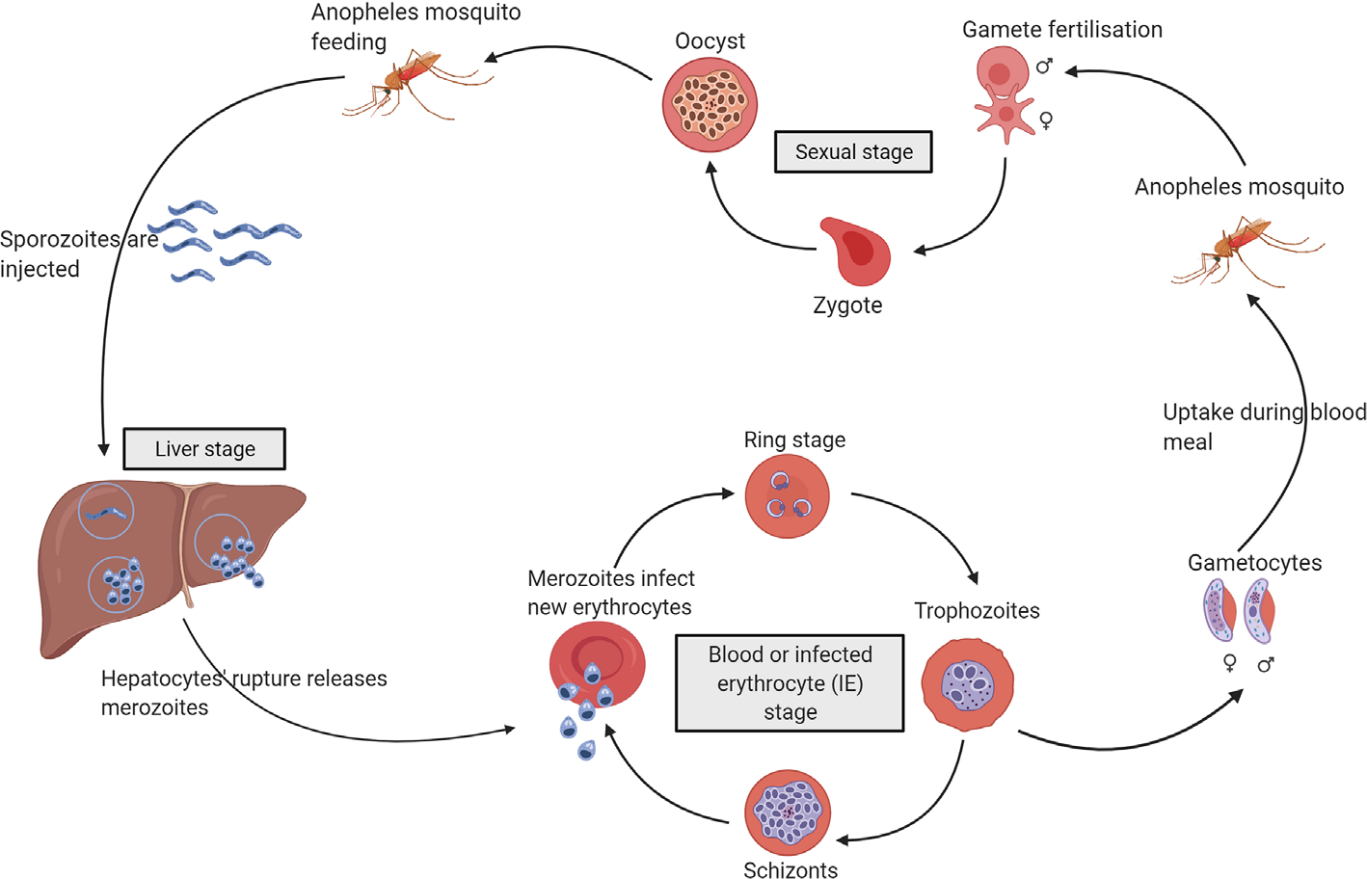
The life cycle of *Plasmodium falciparum. P. falciparum* requires two hosts to complete its life cycle, the mosquito, and the human. Gametocytes are ingested by a female Anopheles mosquito during a blood meal. The gametocytes transform into gametes that fertilise to form zygotes and migrate through the mosquito gut wall to form oocysts. The oocysts rupture and release sporozoites that reach the salivary gland of the mosquito ready to be transmitted into another human host. The injected sporozoites reach the liver, and within hepatocytes, the parasites undergo division (liver stage) before rupture to release merozoites into the bloodstream. The merozoites infect new erythrocytes to form ring-stage parasites that mature into trophozoites and schizonts within the infected erythrocytes (IEs). The rupture of schizonts releases a new generation of merozoites to infect erythrocytes (blood stage). A small proportion of these parasites develop into gametocytes within the IEs, which are ingested by a female Anopheles mosquito for the continuation of the life cycle [reviewed in (3)].

The asexual replication of *Plasmodium* parasites within human erythrocytes is responsible for clinical symptoms of malaria. The parasite has a complex life cycle initiated by a *Plasmodium*-infected mosquito bite (see **Figure 1** for life cycle of *P. falciparum*).

We focus on the importance of understanding the roles of antibody-mediated complement activation in these different stages of the *Plasmodium* life cycle, together with the mechanisms that parasites adopt to evade complement attack to promote their survival. A deeper understanding of antibody-mediated complement activation across the *Plasmodium* life cycle will provide insights into harnessing complement activation in antibody-mediated protection in malaria.

## Complement Activation and Its Role in Malaria Immunity

### Introduction to the Complement System

**Figure 2:**
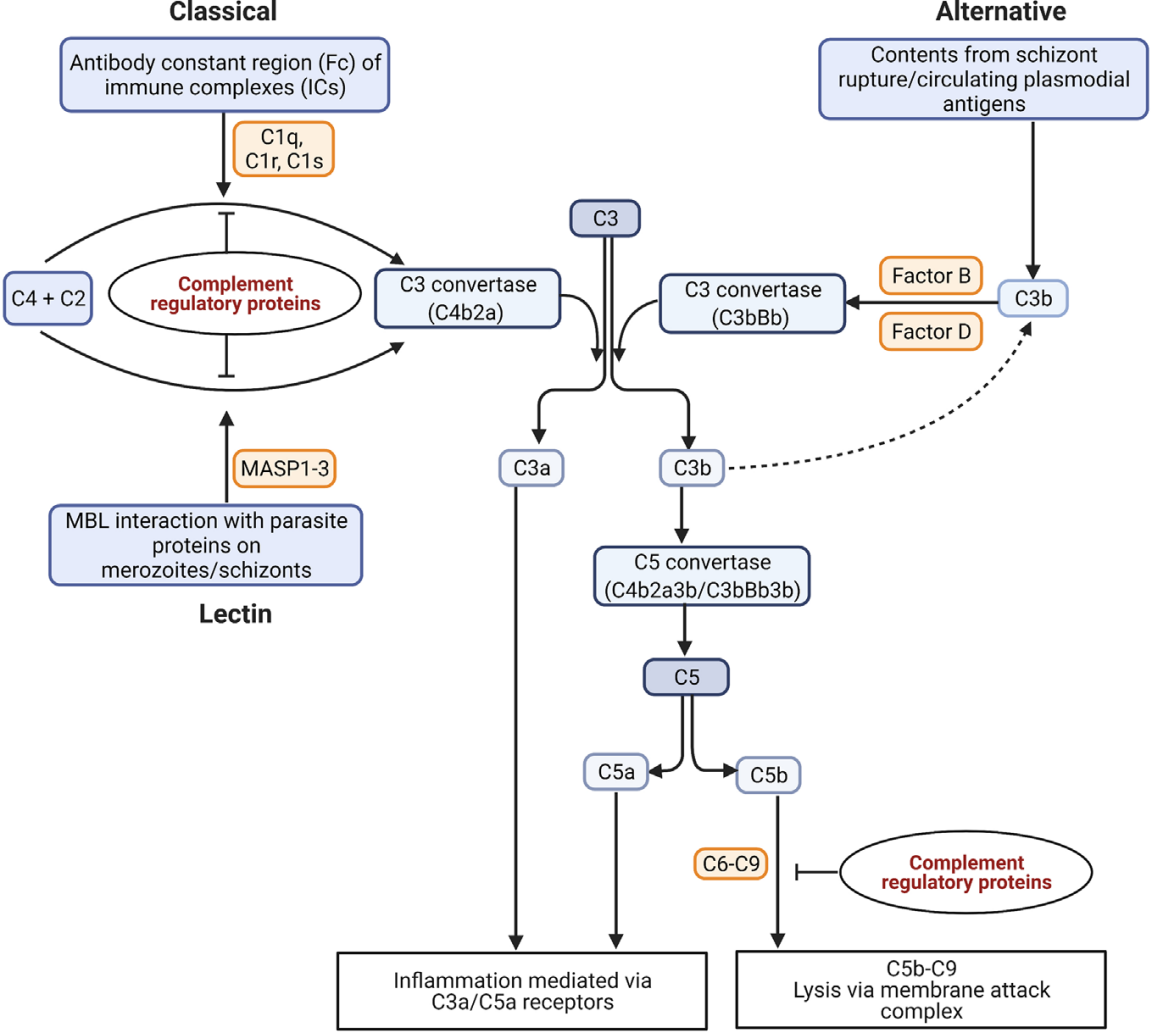
Possible mechanisms of complement activation in malaria. The binding of C1q to antigen-antibody immune complexes (ICs) activates the classical pathway. The lectin pathway can be activated through the binding of mannose with the parasite proteins expressed on schizont surface. The serine proteases MASP1 and MASP2 (Mannan-binding lectin-associated serine proteases 1 and 2) bind to MBL in lectin pathway, and C1r and C1s bind to C1q to cleave two inactive proenzymes C4 and C2 in serum to produce C3 convertase (C4b2a). The alternative pathway is activated either by circulating plasmodial antigens, or products of schizont rupture like hematin. The spontaneous hydrolysis of C3 leads to the cleavage of factor B in serum by an active serum protease called factor D to form a distinct C3 convertase of the alternative pathway (C3bBb). The C3 convertases cleave C3 into C3a and C3b. The products of C3 and C5 cleavage, C3a and C5a, act as anaphylatoxins and interact with immune cell receptors (C3aR and C5aR) to drive inflammation. The assembly of C3b with the C3 convertases produces a C5 convertase (C4b2a3b/C3bBb3b) that can cleave C5 into C5a and C5b. The C5b recruits the factors C6, C7, C8, and C9 for the assembly of the membrane attack complex (MAC) (C5b-C9 or terminal complement complex) for cytolysis (14). [Figure adapted from (15)].

The complement system is a first line of defense against invading pathogens. It consists of both soluble and membrane-bound proteins, of which many are proteases that are proteolytically cleaved in a sequential cascade during activation [reviewed in (11, 12)]. These proteins can be deposited on the surface of pathogens or on host cells to produce a membrane attack complex (MAC) *via* three pathways, namely the classical, alternative, and mannose-binding lectin (MBL) [reviewed in (11)]. In malaria, the complement system may be activated in response to whole parasites or parasite-derived proteins in the host circulation [reviewed in (13)] (**Figure 2**).

The activation of complement is tightly regulated by both soluble and membrane-bound complement regulatory proteins (CRPs) that act at definitive points of the cascade and that are essential to prevent autologous complement attack (**Figure 2**) [reviewed in (14)]. The membrane-bound CRPs are constitutively expressed on the surface of cells including erythrocytes and cells within organs like the kidney, while the fluid-phase CRPs circulate in the plasma and are recruited onto the cell surface upon requirement [reviewed in (16)].

### Levels of Complement in Serum During Malaria

Alterations in the levels of complement in serum have been reported during malaria (17–22). The studies performed in a *P. lophurae* infected duck model showed decreased serum levels of initial complement proteins, C1, C2, and C4 during infection (23). Reduced levels of C4 in serum in simian malaria have been reported implicating classical complement activation (19). Rhesus monkeys infected with *P. coatneyi* showed decreased serum levels of initial complement proteins, C1, C2, and C4 during schizont rupture (24). Human studies showed reduced serum complement levels in patients with cerebral malaria compared to benign infection also indicating classical complement activation (17). Similarly, studies in malaria- infected pregnant women showed increased amounts of C1q, C3d, C4, and C9 in malaria-infected placentas compared to non-infected placentas (25), and deposition of IgG and C3 in some of the *P. falciparum*-infected placentas (26), although no association was shown between infection severity and the amount of complement deposited on the infected placentas. Genome-wide expression analyses showed an upregulation of C1q, C3, C5aR, and C3aR genes in the placentas of primigravid women with malaria compared to placentas of primigravid women without placental malaria (27), implying a role for classical complement activation in disease pathology.

Both the alternative and the classical complement pathways are activated in malaria, indicated by increased levels of alternative pathway derived components, Bb (a breakdown product of factor B) and classical pathway components like C4d (a split product of inactive C4b) as well as soluble C5b-C9 in natural *P. falciparum* infection (28). Complement activation is also regarded as one of the earliest immune responses against experimental *P. falciparum* infection and can be demonstrated when parasitaemia is still undetectable in peripheral blood (22). In studies performed in children with severe malarial anaemia and uncomplicated malaria, the complement system is activated, but a higher level of complement consumption was observed in children with severe malarial anaemia compared to uncomplicated malaria (20).

The parasite components that directly activate complement in malaria include malarial antigens expressed on the surface of IEs (29), and the products of IE rupture like hematin (30). The antigens released by *P. falciparum* growing in culture including merozoites can activate all three pathways of complement, but they cause greatest activation of the alternative pathway (see **Figure 2**) (31).

The role of lectin complement pathway in malaria is not clearly demonstrated. MBL seems to recognise parasite proteins of *P. falciparum*-IEs (32, 33) and may activate lectin pathway.

Genetic studies also reveal a role of MBL protein in malaria. The concentration of MBL protein in serum is genetically determined and different haplotype variants of the MBL gene influence the circulating levels of MBL protein (34). In a study from Gabon, MBL gene polymorphisms were associated with reduced serum levels of MBL protein, and these mutations were present at a higher frequency in children with severe malaria compared to those with mild malaria, suggesting that low MBL levels might be a risk factor for severe malaria (35). This observation was further supported by another study from Ghana that showed low levels of MBL associated with the *mbl2* gene variant increased both susceptibility to *P. falciparum* infection and to severe malaria in young children (36).

By contrast, some studies were unable to show any associations between MBL polymorphisms with parasitaemia and severe disease. Multiple variant alleles of the *mbl2* gene that predicted low serum levels of MBL were not associated with infection or malaria severity in Ghanaian children (33), asymptomatic *P. falciparum* infection in Gabonese children (37), or clinical malaria in Gambian children (38). These discrepancies may be a result of the differences in study design as well as the disease manifestations, and age groups of children enrolled in each study. Taken together, all these studies highlight the possible importance of the lectin pathway in malaria severity, but further studies are needed to fully elucidate the role of MBL and its polymorphisms in malaria susceptibility.

### Antibodies Activate the Classical Complement Pathway in Malaria

**Figure 3:**
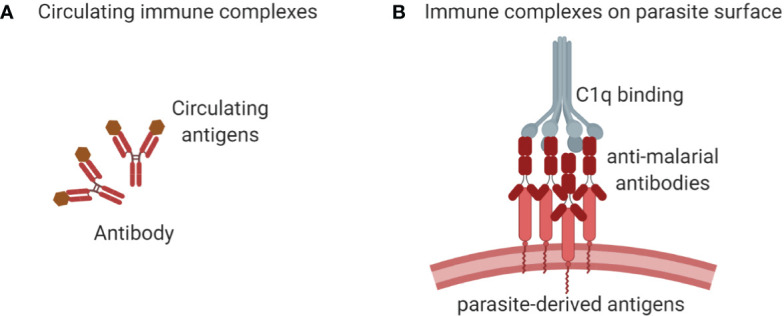
The immune complexes (ICs) are formed when antigens and antibodies unite. They can be formed when the circulating plasmodial antigens cross-link with antibodies in the plasma **(A)** or antibodies can bind with the antigens expressed on the surface of sporozoites, merozoites, or IEs as shown in **(B)**. The circulating ICs (as in A) can also get deposited on the surface of the parasite or on other cells like uninfected erythrocytes, promoting complement deposition on host cell surfaces. These ICs recruit C1q when the globular head domains of C1q bind with the Fc constant region **(B)** of ICs to activate a cascade of downstream events of the classical complement pathway.

Antigen-antibody immune complexes (ICs) activate complement *via* the classical pathway in malaria [reviewed in (39)]. They are formed when antibodies bind malarial antigens circulating in plasma (**Figure 3A**) or expressed on the surface of sporozoites, merozoites, and IEs (**Figure 3B**). In individuals infected with malaria for the first time, ICs may first form approximately 10-14 days after infection, while in subsequent Plasmodium infections complement appears to be activated earlier (13).

The binding of globular head domains of the complement factor C1q with the antibody constant (Fc) domain regions of IgG hexamers or IgM binding antigen activates the classical complement pathway (40). The ability of IgG or IgM antibodies to activate the classical complement pathway depends on antibody isotype and subclass, with IgM-bound ICs having the highest ability to bind C1q and activate the classical pathway, while IgG4 has the lowest activity, and for the other IgG subclasses, the affinities for C1q are IgG3 > IgG1 > IgG2 [reviewed in (41)].

In the next section, we provide a brief overview of antibody-mediated complement fixation on different stages of *P. falciparum* within the human host. We discuss the mechanisms of clearance of the parasites *via* complement-mediated lysis that are brought about by activation of the classical complement pathway in the presence of ICs. We also review the influence of intrinsic and extrinsic properties of both host and parasite that could have a potential impact on complement-dependent lysis of different parasitic stages.

## Antibody-Dependent Complement Fixation on Different Parasitic Stages

### Sporozoites

After injection by the mosquito, *Plasmodium* spp. sporozoites enter the blood vessels and move through the circulation and invade hepatocytes, where they divide to produce merozoites which are released to initiate blood-stage infection (**Figure 1**) [reviewed in (42)]. Targeting sporozoites is potentially an efficient way of preventing malaria as only a small number of sporozoites are injected by the female mosquito during a blood meal.

Studies in murine models have shown that the passive transfer of monoclonal antibodies against the sporozoites of *P. yoelii* inhibited liver infection and the progression to blood-stage infection (43, 44) while in humans, antibodies against *P. falciparum* sporozoites inhibited the movement of sporozoites into hepatocytes *in vitro* (9).

Recent *in vitro* studies conducted in humans showed that these naturally-acquired antibodies against sporozoites of *P. falciparum* can fix complement on the sporozoite surface and are predominantly of cytophilic subclasses, immunoglobulin 1 (IgG1) and IgG3 (9). The hepatocyte transversal inhibitory activity of the naturally acquired anti-sporozoite antibodies was substantially enhanced in the presence of complement, resulting in fixation of C1q on sporozoites and causing their death (9).

Studies show that immunisation with live-attenuated sporozoites of *P. falciparum* can induce sporozoite-specific IgG and IgM antibodies that can fix complement on the sporozoite surface. These antibodies can inhibit sporozoite traversal and invasion into hepatocytes and enhance sporozoite membrane permeability, resulting in sporozoite lysis (45, 46). Similarly, the RTS,S vaccine is based on the major circumsporozoite protein (CSP) on *P. falciparum* sporozoites, and anti-CSP antibodies are induced following RTS,S vaccination that are functional and fix complement factor C1q (47).

In summary, induction of complement-fixing antibodies against sporozoite antigens *via* natural exposure and vaccination can inhibit sporozoite transversal into liver hepatocytes leading to their lysis and death (9), and these antibodies are associated with protection from clinical malaria (9, 46).

### Merozoites

Studies have identified merozoite surface proteins (MSP) such as MSP1_19_, MSP3, and apical membrane antigen-1 [reviewed in (48)], as important targets of protective antibodies, particularly of type IgG (49). The antibodies targeting merozoites limit parasite replication and inhibit invasion of erythrocytes.

Both naturally acquired (50) and vaccine-induced (50, 51) human antibodies against merozoites promote C1q complement deposition on the merozoite surface and activation of the classical complement pathway, inhibiting merozoite invasion and lysing merozoites (50). A longitudinal cohort study performed in older children showed strong associations between complement-fixing antibodies against MSP1 and MSP2 with protection from clinical malaria and high parasitaemia (50). This observation is further supported by a similar study in malaria-exposed children (52) that showed that complement-fixing antibodies against merozoite antigens were a strong predictor of protection against malaria in children.

### Gametocyte-Infected Erythrocytes

Gametocyte-IEs are the infective stages of the parasite that enable transmission of infection from human to mosquito [reviewed in (53)]. There is limited recognition of gametocyte-IEs by naturally acquired antibodies within the human host and this low level of recognition may facilitate the evasion of host immunity and transmission of infection to the mosquito (54).

When an Anopheles mosquito takes a blood meal, host serum components like complement proteins and antibodies are taken in along with the gametocyte-IEs (**Figure 1**) [reviewed in (53)]. In the mosquito midgut, the gametocytes emerge from the erythrocytes, and are exposed to complement proteins and antibodies [reviewed in (53)].

Most studies on immunity to *P. falciparum* sexual stages revolve around the major gametocyte surface antigen, Pfs230 [reviewed in (55)]. Previous studies showed that the transmission blocking activities of monoclonal antibodies against Pfs230 were complement dependent (56), and *in vitro* complement-mediated lysis of gametes by immune sera is associated with antibodies towards Pfs230 (57). But in membrane feeding assays, the transmission blocking activity of immune sera has yet to be shown to be complement dependent (58). Pfs230 is a leading candidate for transmission-blocking vaccines (59), and is likely a major antigenic target for complement-fixing antibodies.

### Infected Erythrocytes

The clinical symptoms of malaria are attributable to blood-stage infection. Some of the major targets of acquired immunity to blood-stage infection are the surface antigens on *P. falciparum-*IEs (60).

Parasite antigens on the surface of IEs play a major role in the pathology of severe malaria *via* parasite sequestration leading to cytoadhesion of IEs to vascular endothelium [reviewed in (61)] or syncytiotrophoblast of the placenta (62). These surface antigens can undergo antigenic variations (63) and are known as variant surface antigens (VSA) (64). The dominant VSAs that are expressed on the surface of IEs are the *P. falciparum* erythrocyte membrane protein 1 (PfEMP1) family of proteins (65).

The surface of trophozoite-IE of *P. falciparum* is a target for antibody-dependent complement activation (29). In the presence of immune sera the classical complement pathway was activated as indicated by formation of complexes of C1s and C1 inhibitor, measured by ELISAs, although antibody-mediated lysis of IEs was not observed visually (29). Spectrometric quantification of the changes in optical absorbance induced by the release of haemoglobin serves as a better option (66).

Later, Weisner et al. showed that the complement cascade can be activated on the surface of IEs, detecting immunoglobulins, C3, C4, and C9 on the surface of IEs (but not uninfected erythrocytes) incubated with immune sera *via* western blot analyses (67). However, they failed to observe IE lysis by classical complement activation in the presence of immune sera suggesting that IEs are resistant to complement-mediated lysis, discussed in more detail in section 5.

Notwithstanding the resistance of antibody-opsonised IEs to complement-mediated lysis, IEs are susceptible to other mechanisms of antibody-mediated removal that are briefly discussed here. Previous studies have shown that antibody-opsonised IEs are cleared by monocytes (68, 69) and neutrophils (70) by cellular phagocytosis. Antibody-opsonised IEs can also activate NK cells, which resulted in lysis of IEs and inhibition of parasite growth (71).

## Complement Activation and Disease Outcomes

### Mechanisms of Immune Complex Clearance

Under normal physiological conditions, the ICs are efficiently cleared by a functional complement system preventing excess deposition of complement that brings detrimental effects to the host [reviewed in (11)]. The complement receptor 1 (CR1) on the surface of macrophages, B cells, neutrophils, dendritic cells, and erythrocytes in humans can recognise complement fragments C3b, iC3b, and C4b that are bound with ICs [reviewed in (11)]. CR1 binds, transports, and endocytoses C3b-bearing ICs to remove them from circulation [reviewed in (14)]. Additionally, the complement receptor 3 (CR3 or CD11b/CD18 complex) on the surface of leukocytes is involved in C3b-mediated opsonic phagocytosis by monocytes and neutrophils and plays a role in clearance of C3b-containing ICs [reviewed in (14)]. The Fc receptors of innate immune cells like neutrophils and monocytes can directly bind to the Fc domain of the ICs also promoting antibody-mediated opsonic phagocytosis (72).

### Complement Activation in the Pathogenesis of Malaria

Mice infected with *P. yoelii* showed a downregulation of the level of expression of CR1 on monocytes or macrophages, a similar though less pronounced downregulation was reported in patients infected with *P. falciparum* and *P. vivax* compared to non-infected controls (73). Decreased CR1 expression on monocytes or macrophages in the mice was also associated with inhibited uptake of immune complexes and was also seen following exposure to lipopolysaccharide (73). Inflammation may contribute to decreased CR1 expression, which then leads to impaired ICs clearance in malaria, and possibly to disease as ICs are associated with increased disease severity (74). But the contribution of ICs to malaria pathogenesis is not fully known.

Both IgG and complement are shown to be deposited on the surface of uninfected erythrocytes in severe malarial anaemia (75, 76). Complement deposition promotes erythrophagocytosis of IC-deposited erythrocytes *via* CR1 on macrophage surface (77). In *Plasmodium* infection, erythrophagocytosis by macrophages seems complement-dependent (78), a possible mechanism for severe malarial anaemia not broadly discussed here (see **Box 1**).

Box 1 **Suggested future research on antibody and complement interactions in malaria.**

- Clarify the cooperation between opsonins C3b and antibody in phagocytosis of merozoites, sporozoites, and IEs by phagocytic cells.
- Clarify the combined roles of both opsonins C3b and antibody in leukocyte activation by merozoites, sporozoites, and IEs.
- Quantify the contribution of C3b deposition on uninfected erythrocytes to anaemia during malaria.
- Investigate the association of complement in disease pathology of cerebral and placental malaria in human and consider whether temporary blockade of C5a generation by complement inhibitors such as the monoclonal anti-C5 antibody eculizumab or the C3 inhibitor compstatin could have a beneficial effect in treatment of cerebral or placental malaria.
- Define other antigenic targets on the surface of merozoites, sporozoites, IEs and gametocytes (if present) that would generate complement-fixing antibodies for protective immunity.
- Determine the association between antibody Fc region variations and antibody functionality for optimum complement activation by opsonised *Plasmodium* antigens.

Other than erythrophagocytosis, erythrocytes with C3b-containing ICs are taken up by splenic reticuloendothelial cells. This may lead to stripping off of the CR1 from the erythrocyte surface (76, 79, 80). CR1-deficient erythrocytes are recirculated and are susceptible to complement attack, implicating complement deposition as a driver of severe malarial anaemia. Among heavily malaria-exposed Gambian children, about half developed a positive direct antiglobulin (Coombs) test (81). IgG was eluted from the surface of their uninfected erythrocytes, and in many cases it was shown to recognise schizonts (82), although the antigen specificity of the antibodies bound to uninfected erythrocytes has not been studied in detail. More recent studies [reviewed in (83)] confirm the deposition of IgG and C3 on these cells. The antibodies are postulated to be immunologically unrelated to the uninfected erythrocytes (81).

Complement activation generates C5a *via* the cleavage of C5, by C5 convertase (see **Figure 2** for complement pathway). C5a is a potent inflammatory mediator (anaphylatoxin) that is readily cleared from plasma *via* receptor internalisation under normal physiological conditions [reviewed in (84)]. The ligation of C5 with C5a receptors (CD88 and C5L2) on innate immune cells promotes a cascade of conventional inflammatory events including increased leukocyte extravasation, neutrophil chemotaxis, degranulation, delayed apoptosis, phagocytosis, oxidative burst, and the activation of vascular endothelial cells to upregulate the expression of cell adhesion molecules [reviewed in (84)].

C5a is implicated in the pathogenesis of cerebral malaria (85) and placental malaria (86, 87). C5a is increased in women with placental malaria (87) and elevated C5a was associated with increased risk of birth of ‘small-for-gestational-age’ babies (86). Blocking C5a in a murine model of placental malaria resulted in improved placental and foetal development (86). Murine models have also highlighted a possible role for C5a in cerebral malaria as C5 deficient mice or those treated with antibodies blocking C5a and its receptor respectively did not develop and could be rescued from cerebral malaria (85). Though there is some evidence for a role of the inflammatory complement C5a protein in disease, the anti-C5 monoclonal antibody eculizumab has not been studied in malaria (see **Box 1**).

The inflammatory effector functions mediated by the release of C5a in response to infection and the C3b-mediated opsonic phagocytosis of IEs (and/or other parasite stages, such as sporozoites, merozoites, and intraerythrocytic gametocytes) by innate immune cells are not discussed in detail in this review.

## Mechanisms of Evasion of Complement-Mediated Lysis by *Plasmodium* IEs

Irrespective of the exposure of blood-stage parasites to serum complement over a relatively prolonged asexual blood-stage (**Figure 1**) (88), the IEs seem to be broadly resistant to complement-mediated lysis (67).

**Figure 4:**
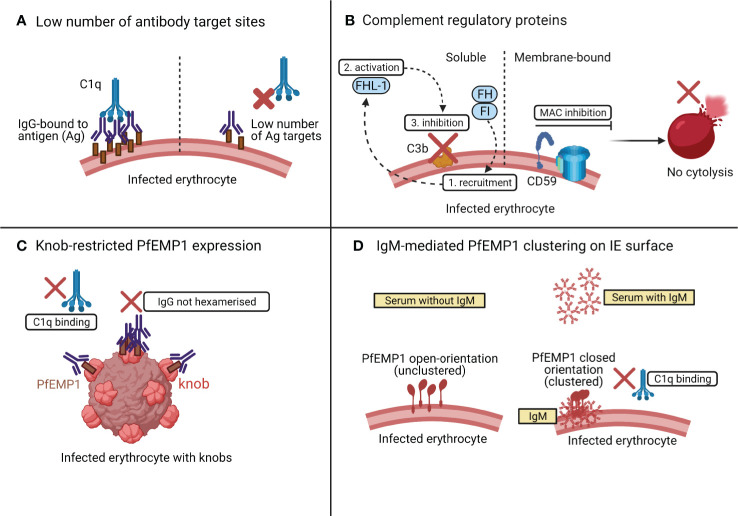
Possible mechanisms of resistance of IEs to classical complement attack. As shown in **(A)**, if the number of PfEMP1 target sites on IEs are below a certain threshold level, complement is not activated even in the presence of an ample supply of antibody and complement. In **(B)**, the presence of complement regulatory proteins such as the membrane bound CD59, inhibits the MAC assembly, to prevent lysis of IEs. The soluble complement regulatory factors [factor I (FI) and factor H (FH)] are also recruited onto the schizont surface (1) and activate FH-like protein 1 (FHL-1) (2) to prevent C3b deposition (3) on the schizont surface, thus preventing complement activation. The knob-restricted expression of PfEMP1 on IEs in **(C)** provides an evolutionary advantage to the parasite to evade classical complement attack. In **(D)**, both PfEMP1 and C1q compete for the same binding site on IgM. Initial binding of IgM with PfEMP1 causes PfEMP1 clustering and prevents IgM-C1q interaction.

One reason why the IEs are resistant to complement-mediated lysis may be that the IEs may have low number of target sites for antibody-binding and complement activation. If this is below a certain threshold, even an excess of antigen-specific antibodies and serum complement may not activate the classical complement pathway (**Figure 4A**) (89).

Membrane-bound CRPs that act at different phases of the complement cascade tightly regulate complement activation [reviewed in (14)]. These include CD46 or membrane cofactor protein (MCP); CR1 which targets and cleaves C3 convertase; CD55 or decay accelerating factor (DAF) which accelerates the decay of both C3 and C5 convertase (90); and CD59 which acts on the terminal complement pathway (see **Figure 2**), targeting C5b-C9 to inhibit the assembly of MAC (90). Interestingly, the IEs appear to have high expression of CD59 that makes them resistant to complement mediated destruction (**Figure 4B**) (67). In addition to the membrane-bound CRPs, the *P. falciparum*-IEs also utilise soluble complement factors like factor I (FI) and factor H (FH) (**Figure 4B**) to evade complement mediated lysis. FH utilises factor I for recruiting FH-related protein FHL-1 onto the schizont surface to inactivate C3b and regulates alternative complement activation in response to the rupture of IEs (91–93).

In an *ex vivo* study of subjects with severe malaria anaemia, the levels of expression of CRPs were lower on uninfected erythrocytes than on IEs (94). This loss of CRPs on uninfected erythrocytes in severe malaria anaemia was not associated with changes in complement-fixing cytophilic antibodies or serum markers of complement activation (as measured by the serum levels of C3a and C5a) (94). High levels of CRPs on IEs may help protect parasites from complement-mediated damage even in the presence of complement-fixing antibodies (94) and even when the terminal complement complexes are deposited on the erythrocyte surface (67).

A recent study assessed the classical complement activation by PfEMP1-specific human IgG using recombinant PfEMP1 by ELISAs and native PfEMP1 on the surface of IEs by flow cytometry (89). The PfEMP1-specific antibodies were unable to activate classical complement pathway by binding to the native PfEMP1 expressed on the surface of IEs (89), but when they bound to recombinant PfEMP1, they activated the classical complement pathway as measured by the elevated levels of C1q, C3, and C4. The authors hypothesised that the knob-restricted expression of native PfEMP1 protein on the IE surface may provide an evolutionary advantage to the parasite to evade classical complement attack (89). The knobs are nanoscale protrusions of the erythrocyte membrane (95) and enable anchorage of PfEMP1 to the surface of IEs (61). The knob-restricted expression of PfEMP1 may hinder the interaction between PfEMP1 and IgG, which may restrict the formation of IgG hexamers for C1q recruitment (40) (**Figure 4C**).

Most of the studies discussed previously highlighted that complement fixing antibodies on IEs were of type IgG (29), especially the subclasses, IgG1 and IgG3 (67). A recent study assessed the complement activation by nonimmune IgM when bound to PfEMP1 on IEs (**Figure 4D**) (96). The binding of IgM to PfEMP1 did not result in complement-mediated lysis because C1q seemed to compete for the same binding pocket on IgM where PfEMP1 is already bound. The interaction between IgM and PfEMP1 prevents C1q deposition and changes the conformation of PfEMP1 to augment PfEMP1-mediated parasite interactions with host receptors for its sequestration and survival (96).

The above reasons seem to contribute to the lack of lysis of IEs by the classical pathway of complement.

## Future Directions and Concluding Remarks

Some evidence (9) suggests that ability to fix complement is an independent correlate of ability of sera to kill sporozoites. Passive transfer studies of a modified monoclonal antibody against PfRh5 (*P. falciparum* reticulocyte homologue 5) indicate that neutralising antibody alone requires high titres (97), indicating a role for Fc-mediated antibody function. This may not, however, be directly attributable to complement fixation as studies of vaccine-induced immunity suggested that NK cell and Fc receptor engagement rather than complement fixation were independent correlates of protection (10).

Despite such recent improvements in understanding of antibody-mediated complement interactions in protective immunity to malaria, the following gaps also should be addressed as priorities for future research (**Box 1**).

In this review, we summarise antibody-dependent complement activation by different stages of parasites during *P. falciparum* infection. Studies show that the inhibitory effect of antibodies directed against surface proteins of sporozoites (e.g., CSP), merozoites (e.g., MSP1_19_), and sexual stages of *P. falciparum* (e.g., Pfs230) can be greatly augmented by the presence of complement. The binding of C1q to IgG-opsonised merozoites and sporozoites has shown to be associated with protective immunity in malaria. We also emphasise that IEs are resistant to complement mediated lysis in comparison to other parasitic stages. This may be due to some intrinsic properties of IEs, including the expression of complement regulatory proteins, an insufficient number of antigenic sites as targets for antibody binding, and the orientation of antigens on the parasite surface for restricted antibody binding for complement deposition. The attempts made to evaluate the underlying mechanisms of antibody-mediated complement activation during malaria will provide a deeper understanding of the processes that mediate complement-mediated protection and/or evasion.

## Author Contributions

DR gathered papers, organised, and drafted the review. Both EA and SR provided intellectual feedback, critically revised, and approved the final manuscript for submission. All authors contributed to the article and approved the submitted version.

## Funding

DR is supported by the Melbourne Research Scholarship from the University of Melbourne and Miller Foundation Top-up Scholarship. The work of EA and SR is supported by grants from the National Health and Medical Research Council of Australia (GNT1092789 and GNT1143946), and by the Centre for Research Excellence in Malaria Elimination (GNT1134989).

## Conflict of Interest

The authors declare that the research was conducted in the absence of any commercial or financial relationships that could be construed as a potential conflict of interest.

## References

[1] World Health Organization. World Malaria Report 2020. In: 20 Years of Global Progress and Challenges. Geneva: World Health Organization. (2020).

[2] Langhorne J Ndungu FM Sponaas AM Marsh K. Immunity to Malaria: More Questions Than Answers. Nat Immunol (2008) 9(7):725–32. 10.1038/ni.f.205 18563083

[3] Ashley EA Pyae Phyo A Woodrow CJ. Malaria. Lancet (2018) 391:1608–21. 10.1016/S0140-6736(18)30324-6 29631781

[4] Desai M ter Kuile FO Nosten F McGready R Asamoa K Brabin B. Epidemiology and Burden of Malaria in Pregnancy. Lancet Infect Dis (2007) 7(2):93–104. 10.1016/S1473-3099(07)70021-X 17251080

[5] Basu S Sahi PK. Malaria: An Update. Indian J Pediatr (2017) 84(7):521–8. 10.1007/s12098-017-2332-2 28357581

[6] Doolan DL Dobaño C Baird JK. Acquired Immunity to Malaria. Clin Microbiol Rev (2009) 22(1):13–36. 10.1128/CMR.00025-08 19136431 PMC2620631

[7] Cohen S McGregor IA Carrington S. Gamma-Globulin and Acquired Immunity to Human Malaria. Nature (1961) 192:733–7. 10.1038/192733a0 13880318

[8] Kurtovic L Atre T Feng G Wines BD Chan J-A Boyle MJ. Multifunctional Antibodies Are Induced by the RTS,S Malaria Vaccine and Associated With Protection in a Phase 1/2a Trial. J Infect Dis (2020) jiaa144:1–11. 10.1093/infdis/jiaa144 PMC8514181 32236404

[9] Kurtovic L Behet MC Feng G Reiling L Chelimo K Dent AE. Human Antibodies Activate Complement Against Plasmodium Falciparum Sporozoites, and are Associated With Protection Against Malaria in Children. BMC Med (2018) 16(1):1–17. 10.1186/s12916-018-1054-2 PMC5925837 29706136

[10] Suscovich TJ Fallon JK Das J Demas AR Crain J Linde CH. Mapping Functional Humoral Correlates of Protection Against Malaria Challenge Following RTS,S/AS01 Vaccination. Sci Transl Med (2020) 12(553):1–14. 10.1126/scitranslmed.abb4757 32718991

[11] Ricklin D Hajishengallis G Yang K Lambris JD. Complement: A Key System for Immune Surveillance and Homeostasis. Nat Immunol (2010) 11(9):785–97. 10.1038/ni.1923 PMC2924908 20720586

[12] Wagner E Frank MM. Therapeutic Potential of Complement Modulation. Nat Rev Drug Discovery (2010) 9(1):43–56. 10.1038/nrd3011 19960015

[13] Taylor RP Stoute JA Lindorfer MA. Mechanisms of Complement Activation in Malaria. In: Stoute JA, editor. Complement Activation in Malaria Immunity and Pathogenesis. United States: Springer (2018). p. 31–49.

[14] Merle NS Church SE Fremeaux-Bacchi V Roumenina LT. Complement System Part I - Molecular Mechanisms of Activation and Regulation. Front Immunol (2015) 6:1–30. 10.3389/fimmu.2015.00262 26082779 PMC4451739

[15] Nauser CL Farrar CA Sacks SH. Complement Recognition Pathways in Renal Transplantation. J Am Soc Nephrol (2017) 28(9):2571–8. 10.1681/ASN.2017010079 PMC5576943 28663231

[16] Noris M Remuzzi G. Overview of Complement Activation and Regulation. Semin Nephrol (2013) 33(6):479–92. 10.1016/j.semnephrol.2013.08.001 PMC3820029 24161035

[17] Adam C Grniteau M Gougerot-Pocidalo P Verroust P Lebras J Gibert C. Cryoglobulins, Circulating Immune Complexes, and Complement Activation in Cerebral Malaria. Infect Immun (1981) 31(2):530–5. 10.1128/IAI.31.2.530-535.1981 PMC351340 7012010

[18] Ade-Serrano MA Ejezie GC Kassim OO. Correlation of Plasmodium Falciparum Gametocytemia With Complement Component Titers in Rural Nigerian School Children. J Clin Microbiol (1981) 13(1):195–8. 10.1128/JCM.13.1.195-198.1981 PMC273747 7007423

[19] Glew RH Atkinson JP Frank MM Collins WE Neva FA. Serum Complement and Immunity in Experimental Simian Malaria. I. Cyclical Alterations in C4 Related to Schizont Rupture. J Infect Dis (1975) 131(1):17–25. 10.1093/infdis/131.1.17 1089126

[20] Nyakoe NK Taylor RP Makumi JN Waitumbi JN. Complement Consumption in Children With Plasmodium Falciparum Malaria. Malar J (2009) 8(1):7. 10.1186/1475-2875-8-7 19134190 PMC2645421

[21] Neva FA Howard WA Glew RH Krotoski WA Gam AA Collins WE. Relationship of Serum Complement Levels to Events of the Malarial Paroxysm. J Clin Invest (1974) 54(2):451–60. 10.1172/JCI107781 PMC301573 4603170

[22] Roestenberg M McCall M Mollnes TE van Deuren M Sprong T Klasen I. Complement Activation in Experimental Human Malaria Infection. Trans R Soc Trop Med Hyg (2007) 101(7):643–9. 10.1016/j.trstmh.2007.02.023 17481680

[23] McGhee RB. The Effect of a Malaria Infection on the Titer of Complement and its Components in Ducks. J Immunol (1952) 68(4):421–7. 14946382

[24] Atkinson JP Glew RH Neva FA Frank MM. Serum Complement and Immunity in Experimental Simian Malaria. II. Preferential Activation of Early Components and Failure of Depletion of Late Components to Inhibit Protective Immunity. J Infect Dis (1975) 131(1):26–33. 10.1093/infdis/131.1.26 1110307

[25] Galbraith RM Fox H Hsi B Galbraith GMP Bray RS Faulk WP. The Human Materno-Foetal Relationship in Malaria. II. Histological, Ultrastructural, and Immunopathologic Studies of the Placenta. Trans R Soc Trop Med Hyg (1980) 74(1):61–72. 10.1016/0035-9203(80)90012-7 7001685

[26] Yamada M Steketee R Abramowsky C Kida M Wirima J Heymann D. Plasmodium Falciparum Associated Placental Pathology: A Light and Electron Microscopic and Immunohistologic Study. Am J Trop Med Hyg (1989) 41(2):161–8. 10.4269/ajtmh.1989.41.161 2672835

[27] Muehlenbachs A Fried M Lachowitzer J Mutabingwa TK Duffy PE. Genome-Wide Expression Analysis of Placental Malaria Reveals Features of Lymphoid Neogenesis During Chronic Infection. J Immunol (2007) 179:557–65. 10.4049/jimmunol.179.1.557 17579077

[28] Wenisch C Spitzauer S Florris-Linau K Rumpold H Vannaphan S Parschalk B. Complement Activation in Severe Plasmodium Falciparum Malaria. Clin Immunol Immunopathol (1997) 85(2):166–71. 10.1006/clin.1997.4417 9344699

[29] Stanley HA Mayes JT Cooper NR Reese RT. Complement Activation by the Surface of Plasmodium Falciparum Infected Erythrocytes. Mol Immunol (1984) 21(2):145–50. 10.1016/0161-5890(84)90129-9 6369119

[30] Pawluczkowycz AW Lindorfer MA Waitumbi JN Taylor RP. Hematin Promotes Complement Alternative Pathway-Mediated Deposition of C3 Activation Fragments on Human Erythrocytes: Potential Implications for the Pathogenesis of Anemia in Malaria. J Immunol (2007) 179(8):5543–52. 10.4049/jimmunol.179.8.5543 17911641

[31] Korir JC Nyakoe NK Awinda G Waitumbi JN. Complement Activation by Merozoite Antigens of Plasmodium Falciparum. PloS One (2014) 9(8):1–9. 10.1371/journal.pone.0105093 PMC4140736 25144772

[32] Klabunde J Uhlemann A-C Tebo AE Kimmel J Schwarz RT Kremsner PG. Recognition of Plasmodium Falciparum Proteins by Mannan-Binding Lectin, A Component of the Human Innate Immune System. Parasitol Res (2002) 88(2):113–7. 10.1007/s00436-001-0518-y 11936498

[33] Garred P Nielsen MA Kurtzhals JAL Malhotra R Madsen HO Goka BQ. Mannose-Binding Lectin is a Disease Modifier in Clinical Malaria and May Function as Opsonin for Plasmodium Falciparum- Infected Erythrocytes. Infect Immun (2003) 71(9):5245–53. 10.1128/IAI.71.9.5245-5253.2003 PMC187320 12933871

[34] Lipscombe RJ Sumiya M Summerfield JA Turner MW. Distinct Physicochemical Characteristics of Human Mannose Binding Protein Expressed by Individuals of Differing Genotype. Immunology (1995) 85:660–7. PMC1383797 7558163

[35] Luty AJF Kun JFJ Kremsner PG. Mannose-Binding Lectin Plasma Levels and Gene Polymorphisms in Plasmodium Falciparum Malaria. J Infect Dis (1998) 178(4):1221–4. 10.1086/515690 9806066

[36] Holmberg V Schuster F Dietz E Sagarriga JC Anemana SD Bienzle U. Mannose-Binding Lectin Variant Associated With Severe Malaria in Young African Children. Microbes Infect (2008) 10:342–8. 10.1016/j.micinf.2007.12.008 18396436

[37] Mombo LE Ntoumi F Bisseye C Ossari S Lu CY Nagel RL. Human Genetic Polymorphisms and Asymptomatic Plasmodium Falciparum Malaria in Gabonese School Children. Am J Trop Med Hyg (2003) 68(2):186–90. 10.4269/ajtmh.2003.68.186 12641410

[38] Bellamy R Ruwende C Mcadam KPWJ Thursz M Sumiya M Summerfield J. Mannose Binding Protein Deficiency Is Not Associated With Malaria, Hepatitis B Carriage Nor Tuberculosis in Africans. Q J Med (1998) 91:13–8. 10.1093/qjmed/91.1.13 9519208

[39] Biryukov S Stoute JA. Complement Activation in Malaria: Friend or Foe? Trends Mol Med (2014) 20(5):293–301. 10.1016/j.molmed.2014.01.001 24508275

[40] Diebolder CA Beurskens FJ Jong RND Koning RI Strumane K Lindorfer MA. Complement Is Activated by IgG Hexamers Assembled at the Cell Surface. Science (2014) 343(6176):1260–3. 10.1126/science.1248943 PMC4250092 24626930

[41] Schroeder HW Cavacini L. Structure and Function of Immunoglobulins. J Allergy Clin Immunol (2010) 125:237–49. 10.1016/j.jaci.2009.09.046 PMC3670108 20176268

[42] Dundas K Shears MJ Photini S Wright GJ. Important Extracellular Interactions Between Plasmodium Sporozoites and Host Cells Required for Infection. Trends Parasitol (2016) 35(2):1–18. 10.1016/j.pt.2018.11.008 PMC6375296 30583849

[43] Sack BK Miller JL Vaughan AM Douglass A Kaushansky A Mikolajczak S. Model for In Vivo Assessment of Humoral Protection Against Malaria Sporozoite Challenge by Passive Transfer of Monoclonal Antibodies and Immune Serum. Infect Immun (2014) 82(2):808–17. 10.1128/IAI.01249-13 PMC3911395 24478094

[44] Mellouk S Berbiguier N Druilhe P Sedegah M Galey B Leef M. Evaluation of an In Vitro Assay Aimed at Measuring Protective Antibodies Against Sporozoites. Bull World Health Organ (1990) 68:52–9. PMC2393043 2094592

[45] Zenklusen I Jongo S Abdulla S Ramadhani K Lee Sim BK Cardamone H. Immunization of Malaria-Preexposed Volunteers With PfSPZ Vaccine Elicits Long-Lived IgM Invasion-Inhibitory and Complement-Fixing Antibodies. J Infect Dis (2018) 217(10):1569–78. 10.1093/infdis/jiy080 PMC5913598 29438525

[46] Behet MC Kurtovic L van Gemert GJ Haukes CM Siebelink-Stoter R Graumans W. The Complement System Contributes to Functional Antibody-Mediated Responses Induced by Immunization With Plasmodium Falciparum Malaria Sporozoites. Infect Immun (2018) 86(7):1–15. 10.1128/IAI.00920-17 PMC6013677 29735521

[47] Kurtovic L Agius PA Feng G Drew DR Ubillos I Sacarlal J. Induction and Decay of Functional Complement-Fixing Antibodies by the RTS,S Malaria Vaccine in Children, and a Negative Impact of Malaria Exposure. BMC Med (2019) 17(1):1–14. 10.1186/s12916-019-1277-x 30798787 PMC6388494

[48] Cowman AF Crabb BS. Invasion of Red Blood Cells by Malaria Parasites. Cell (2006) 124:755–66. 10.1016/j.cell.2006.02.006 16497586

[49] Stanisic DI Richards JS McCallum FJ Michon P King CL Schoepflin S. Immunoglobulin G Subclass-Specific Responses Against Plasmodium Falciparum Merozoite Antigens are Associated With Control of Parasitemia and Protection From Symptomatic Illness. Infect Immun (2009) 77(3):1165–74. 10.1128/IAI.01129-08 PMC2643653 19139189

[50] Boyle MJ Reiling L Feng G Langer C Osier FH Aspeling-Jones H. Human Antibodies Fix Complement to Inhibit Plasmodium Falciparum Invasion of Erythrocytes and are Associated With Protection Against Malaria. Immunity (2015) 42(3):580–90. 10.1016/j.immuni.2015.02.012 PMC4372259 25786180

[51] Feng G Boyle MJ Cross N Chan JA Reiling L Osier F. Human Immunization With a Polymorphic Malaria Vaccine Candidate Induced Antibodies to Conserved Epitopes That Promote Functional Antibodies to Multiple Parasite Strains. J Infect Dis (2018) 218(1):35–43. 10.1093/infdis/jiy170 29584918 PMC6904323

[52] Reiling L Boyle MJ White MT Wilson DW Feng G Weaver R. Targets of Complement-Fixing Antibodies in Protective Immunity Against Malaria in Children. Nat Commun (2019) 10(1):610. 10.1038/s41467-019-08528-z 30723225 PMC6363798

[53] Jong RM Tebeje SK Meerstein-Kessel L Tadesse FG Jore MM Stone W. Immunity Against Sexual Stage Plasmodium Falciparum and Plasmodium Vivax Parasites. Immunol Rev (2020) Jan 16 293(1):190–215. 10.1111/imr.12828 PMC6973022 31840844

[54] Chan JA Drew DR Reiling L Lisboa-Pinto A Dinko B Sutherland CJ. Low Levels of Human Antibodies to Gametocyte-Infected Erythrocytes Contrasts the PfEMP1-Dominant Response to Asexual Stages in P. Falciparum Malaria. Front Immunol (2019) 9:1–8. 10.3389/fimmu.2018.03126 PMC6340286 30692996

[55] Williamson KC. Pfs230: From Malaria Transmission-Blocking Vaccine Candidate Toward Function. Parasite Immunol (2003) 25:351–9. 10.1046/j.1365-3024.2003.00643.x 14521577

[56] Read D Lensen AH Begarnie Haley S Raza A Carter R. Transmission-Blocking Antibodies Against Multiple, Non-Variant Target Epitopes of the Plasmodium Falciparum Gamete Surface Antigen Pfs230 are All Complement-Fixing. Parasite Immunol (1994) 16:511–9. 10.1111/j.1365-3024.1994.tb00305.x 7532850

[57] Healer J McGuinness D Hopcroft P Haley S Carter R Riley E. Complement-Mediated Lysis of Plasmodium Falciparum Gametes by Malaria- Immune Human Sera is Associated With Antibodies to the Gamete Surface Antigen Pfs230. Infect Immun (1997) 65(8):3017–23. 10.1128/IAI.65.8.3017-3023.1997 PMC175425 9234748

[58] Roeffen W Beckers PJA Teelen K Lensen T Sauerwein RW Meuwissen JHET. Plasmodium Falciparum: A Comparison of the Activity of Pfs230-Specific Antibodies in an Assay of Transmission-Blocking Immunity and Specific Competition Elisas. Exp Parasitol (1995) 80:15–26. 10.1006/expr.1995.1003 7529717

[59] Singh K Burkhardt M Nakuchima S Herrera R Muratova O Gittis AG. Structure and Function of a Malaria Transmission Blocking Vaccine Targeting Pfs230 and Pfs230-Pfs48/45 Proteins. Commun Biol (2020) 3(1):1–12. 10.1038/s42003-020-01123-9 32709983 PMC7381611

[60] Chan J-A Stanisic DI Duffy MF Robinson LJ Lin E Kazura JW. Patterns of Protective Associations Differ for Antibodies to P. Falciparum -Infected Erythrocytes and Merozoites in Immunity Against Malaria in Children. Eur J Immunol (2017) 47(12):2124–36. 10.1002/eji.201747032 PMC5893221 28833064

[61] Smith JD Rowe JA Higgins MK Lavstsen T. Malaria’s Deadly Grip: Cytoadhesion of Plasmodium Falciparum Infected Erythrocytes. Cell Microbiol (2013) 15(12):1–7. 10.1111/cmi.12183 23957661 PMC3836831

[62] Fried M Duffy P. Adherence of Plasmodium Falciparum to Chondroitin Sulfate A in the Human Placenta. Science (1996) 272:2–5. 10.1126/science.272.5267.1502 8633247

[63] Roberts DJ Craig AG Berendt AR Pinches R Nash G Marsh K. Rapid Switching to Multiple Antigenic and Adhesive Phenotypes in Malaria. Nature (1992) 357(6380):689–92. 10.1038/357689a0 PMC3731710 1614515

[64] Smith JD Craig AG Roberts DJ Hudson-taylor DE Peterson DS Pinches R. Switches in Expression of Plasmodium Falciparum Var Genes Correlate With Changes in Antigenic and Cytoadherent Phenotypes of Infected Erythrocytes. Cell (1995) 82(1):101–10. 10.1016/0092-8674(95)90056-X PMC3730239 7606775

[65] Chan JA Howell KB Reiling L Ataide R Mackintosh CL Fowkes FJI. Targets of Antibodies Against Plasmodium Falciparum-Infected Erythrocytes in Malaria Immunity. J Clin Invest (2012) 122(9):3227–38. 10.1172/JCI62182 PMC3428085 22850879

[66] Meulenbroek EM Wouters D Zeerleder S. Methods for Quantitative Detection of Antibody-induced Complement Activation on Red Blood Cells. J Vis Exp (2014) 83:1–6. 10.3791/51161 PMC4091512 24514151

[67] Wiesner J Jomaa H Wilhem M Tony H Kremsner PG Horrocksl P. Host Cell Factor CD59 Restricts Complement Lysis of Plasmodium Falciparum-Infected Erythrocytes. Eur J Immunol (1997) 27:2708–13. 10.1002/eji.1830271034 9368630

[68] Hommel M Chan J Umbers AJ Langer C Rogerson SJ Smith JD. Evaluating Antibody Functional Activity and Strain-Specificity of Vaccine Candidates for Malaria in Pregnancy Using In Vitro Phagocytosis Assays. Parasit Vectors (2018) 11(69):1–7. 10.1186/s13071-018-2653-7 29378634 PMC5789608

[69] Zhou J Feng G Beeson J Hogarth PM Rogerson SJ Yan Y. Cd14hiCD16+ Monocytes Phagocytose Antibody-Opsonised Plasmodium Falciparum Infected Erythrocytes More Efficiently Than Other Monocyte Subsets, and Require CD16 and Complement to do So. BMC Med (2015) 13(1):1–14. 10.1186/s12916-015-0391-7 26149666 PMC4493812

[70] Celada A Cruchaud A Perrin LH. Phagocytosis of Plasmodium Falciparum-Parasitized Erythrocytes by Human Polymorphonuclear Leukocytes. J Parasitol (1983) 69(1):49–53. 10.2307/3281273 6338199

[71] Arora G Hart GT Manzella-lapeira J Doritchamou JYA Narum DL Thomas LM. NK Cells Inhibit Plasmodium Falciparum Growth in Red Blood Cells Via Antibody- Dependent Cellular Cytotoxicity. Elife (2018) 7:1–20. 10.7554/eLife.36806 PMC6019063 29943728

[72] Nardin A Lindorfer MA Taylor RP. How are Immune Complexes Bound to the Primate Erythrocyte Complement Receptor Transferred to Acceptor Phagocytic Cells? Mol Immunol (1999) 36:827–35. 10.1016/S0161-5890(99)00103-0 10698336

[73] Fernandez-Arias C Lopez JP Nikolae J Bautista-ojeda MD Bautista-ojeda MD Branch O. Malaria Inhibits Surface Expression of Complement Receptor 1 in Monocytes/Macrophages, Causing Decreased Immune Complex Internalization. J Immunol (2013) 190:3363–72. 10.4049/jimmunol.1103812 PMC3673585 23440418

[74] Thomas BN Diallo DA Noumsi GT Moulds JM. Circulating Immune Complex Levels are Associated With Disease Severity and Seasonality in Children With Malaria From Mali. Biomark Insights (2012) 7:81–6. 10.4137/BMI.S9624 PMC3399413 22837639

[75] Owuor BO Odhiambo CO Otieno WO Adhiambo C Makawiti DW Stoute JA. Reduced Immune Complex Binding Capacity and Increased Complement Susceptibility of Red Cells From Children With Severe Malaria-Associated Anemia. Mol Med (2007) 14(3–4):89–97. 10.2119/2007-00093.Owuor PMC2258160 18317566

[76] Waitumbi JN Opollo MO Muga RO Misore AO Stoute A. Red Cell Surface Changes and Erythrophagocytosis in Children With Severe Plasmodium Falciparum Anemia. Blood (2000) 95(4):1481–7. 10.1182/blood.V95.4.1481.004k15_1481_1486 10666228

[77] Goka BQ Kwarko H Kurtzhals JAL Gyan B Ofori-Adjei E Ohene SA. Complement Binding to Erythrocytes Is Associated With Macrophage Activation and Reduced Haemoglobin in Plasmodium Falciparum Malaria. Trans R Soc Trop Med Hyg (2001) 95(5):545–9. 10.1016/S0035-9203(01)90036-7 11706671

[78] Dasari P Fries A Heber SD Salama A Blau I-W Lingelbach K. Malarial Anemia: Digestive Vacuole of Plasmodium Falciparum Mediates Complement Deposition on Bystander Cells to Provoke Hemophagocytosis. Med Microbiol Immunol (2014) 203:383–93. 10.1007/s00430-014-0347-0 24985035

[79] Waitumbi JN Donvito B Kisserli A Cohen JHM Stoute JA. Age-Related Changes in Red Blood Cell Complement Regulatory Proteins and Susceptibility to Severe Malaria. J Infect Dis (2004) 190(6):1183–91. 10.1086/423140 15319870

[80] Stoute JA Odindo AO Owuor BO Mibei EK Opollo MO Waitumbi JN. Loss of Red Blood Cell–Complement Regulatory Proteins and Increased Levels of Circulating Immune Complexes are Associated With Severe Malarial Anemia. J Infect Dis (2003) 187(3):522–5. 10.1086/367712 12552440

[81] Facer CA Bray RS Brown J. Direct Coombs Antiglobulin Reactions in Gambian Children With Plasmodium Falciparum Malaria I. Incidence Class Specificity Clin Exp Immunol (1979) 35:119–27. PMC1537580 371880

[82] Facer CA. Direct Coombs Antiglobulin Reactions in Gambian Children With Plasmodium Falciparum Malaria II. Specificity Erythrocyte-Bound IgG Clin Exp Immunol (1980) 39:279–88. PMC1538045 6993068

[83] Stoute JA. Complement Receptor 1 and Malaria. Cell Microbiol (2011) 13(10):1441–50. 10.1111/j.1462-5822.2011.01648.x 21790941

[84] Wood AJT Vassallo A Summers C Chilvers ER Conway-Morris A. C5a Anaphylatoxin and its Role in Critical Illness-Induced Organ Dysfunction. Eur J Clin Invest (2018) 48(12):e13028. 10.1111/eci.13028 30229880

[85] Patel SN Berghout J Lovegrove FE Ayi K Conroy A Serghides L. C5 Deficiency and C5a or C5aR Blockade Protects Against Cerebral Malaria. J Exp Med (2008) 205(5):1133–43. 10.1084/jem.20072248 PMC2373845 18426986

[86] Conroy AL Silver KL Zhong K Rennie M Ward P Sarma JV. Complement Activation and the Resulting Placental Vascular Insufficiency Drives Fetal Growth Restriction Associated With Placental Malaria. Cell Host Microbe (2013) 13(2):215–26. 10.1016/j.chom.2013.01.010 23414761

[87] Conroy A Serghides L Finney C Owino SO Kumar S Gowda DC. C5a Enhances Dysregulated Inflammatory and Angiogenic Responses to Malaria in Vitro: Potential Implications for Placental Malaria. PloS One (2009) 4(3):e4953. 10.1371/journal.pone.0004953 19308263 PMC2655724

[88] David PH Hommel M Miller LH Udeinya IJ Oligino LD. Parasite Sequestration in Plasmodium Falciparum Malaria: Spleen and Antibody Modulation of Cytoadherence of Infected Erythrocytes. Proc Natl Acad Sci (1983) 80(16):5075–9. 10.1073/pnas.80.16.5075 PMC384191 6348780

[89] Larsen MD Quintana M del P Ditlev SB Bayarri-Olmos R Ofori MF Hviid L. Evasion of Classical Complement Pathway Activation on Plasmodium Falciparum-Infected Erythrocytes Opsonized by PfEMP1-Specific Igg. Front Immunol (2019) 9(Jan):1–10. 10.3389/fimmu.2018.03088 PMC6330326 30666256

[90] Sarma JV Ward PA. The Complement System. Cell Tissue Res (2011) 343(1):227–35. 10.1007/s00441-010-1034-0 PMC3097465 20838815

[91] Rosa TFA Flammersfeld A Ngwa CJ Kiesow M Fischer R Zipfel PF. The Plasmodium Falciparum Blood Stages Acquire Factor H Family Proteins to Evade Destruction by Human Complement. Cell Microbiol (2016) 18(4):573–90. 10.1111/cmi.12535 PMC5063132 26457721

[92] Reiss T Rosa TF de A Blaesius K Bobbert RP Zipfel PF Skerka C. Cutting Edge: Fhr-1 Binding Impairs Factor H–Mediated Complement Evasion by the Malaria Parasite Plasmodium Falciparum. J Immunol (2018) 201(12):3497–502. 10.4049/jimmunol.1800662 30455399

[93] Kennedy AT Schmidt CQ Thompson JK Weiss GE Taechalertpaisarn T Gilson PR. Recruitment of Factor H as a Novel Complement Evasion Strategy for Blood-Stage Plasmodium Falciparum Infection. J Immunol (2016) 196(3):1239–48. 10.4049/jimmunol.1501581 26700768

[94] Oyong DA Kenangalem E Poespoprodjo JR Beeson JG Anstey NM Price RN. Loss of Complement Regulatory Proteins on Uninfected Erythrocytes in Vivax and Falciparum Malaria Anemia. JCI Insight (2018) 3(22):1–11. 10.1172/jci.insight.124854 PMC6303009 30429373

[95] Nagao E Kaneko O Dvorak JA. Plasmodium Falciparum-Infected Erythrocytes: Qualitative and Quantitative Analyses of Parasite-Induced Knobs by Atomic Force Microscopy. J Struct Biol (2000) 130:34–44. 10.1006/jsbi.2000.4236 10806089

[96] Akhouri RR Goel S Furusho H Skoglund U Wahlgren M. Architecture of Human Igm in Complex With P. Falciparum Erythrocyte Membrane Protein 1 Cell Rep (2016) Feb 14(4):723–36. 10.1016/j.celrep.2015.12.067 26776517

[97] Douglas AD Baldeviano GC Jin J Miura K Diouf A Zenonos ZA. A Defined Mechanistic Correlate of Protection Against Plasmodium Falciparum Malaria in non-Human Primates. Nat Commun (2019) 10(1953):1–8. 10.1038/s41467-019-09894-4 31028254 PMC6486575

